# Hypoxia-Induced miR-137 Inhibition Increased Glioblastoma Multiforme Growth and Chemoresistance Through LRP6

**DOI:** 10.3389/fonc.2020.611699

**Published:** 2021-02-25

**Authors:** Dong-Mei Li, Qiu-Dan Chen, Gui-Ning Wei, Jie Wei, Jian-Xing Yin, Jun-Hui He, Xin Ge, Zhu-Mei Shi

**Affiliations:** ^1^ Department of Pharmacology, Guangxi Institute of Chinese Medicine & Pharmaceutical Science, Nanning, China; ^2^ Institute for Brain Tumors, Jiangsu Key Lab of Cancer Biomarkers, Prevention and Treatment, Jiangsu Collaborative Innovation Center for Cancer Personalized Medicine, Nanjing Medical University, Nanjing, China; ^3^ The Department of Central Laboratory, Clinical Laboratory, Jing’an District Center Hospital of Shanghai, Fudan University, Shanghai, China; ^4^ Department of Neurosurgery, The First Affiliated Hospital of Nanjing Medical University, Nanjing, China; ^5^ Department of Nutrition and Food Hygiene, School of Public Health, Nanjing Medical University, Nanjing, China

**Keywords:** glioblastoma multiforme, miR-137, chemoresistance, LRP6, epithelial-mesenchymal transition-related genes

## Abstract

**Purpose:**

Glioblastoma multiforme (GBM) is one of the deadliest tumors, which is involved in numerous dysregulated microRNAs including miR-137. However, the mechanism of how miR-137 suppression associated with cancer progression and chemoresistance still remains to be elucidated.

**Methods:**

Quantitative reverse transcriptase-PCR (qRT-PCR), DNA methylation analysis, cell proliferation assay, flow cytometric analysis, invasion assay, *in situ* tumor formation experiment were performed to test the expression levels and functions of miR-137 in GBM. Bioinformatics analysis, luciferase reporter assay, qRT-PCR, immunoblotting, immunofluorescence, and immunohistochemistry assay were used to identify and verify the target of miR-137.

**Results:**

We found that miR-137 was downregulated in primary and recurrent GBM compared with normal brain tissues. Overexpression of miR-137 inhibited cell invasion and enhanced cell chemosensitivity to temozolomide (TMZ) by directly targeting low-density lipoprotein receptor-related protein 6 (LRP6) in GBM. Forced expression of LRP6 cDNA without its 3’-UTR region partly restored the effects of miR-137 *in vitro* and *in vivo*. Hypoxia-induced miR-137 methylation was responsible for the miR-137 suppression, leading to the cell chemoresistance and poor prognosis of GBM.

**Conclusions:**

These findings demonstrated the detailed molecular mechanism of miR-137 in regulating GBM growth and chemoresistance in hypoxia microenvironment, suggesting the potentiality of miR-137 as a therapeutic target for GBM.

## Background

Glioblastoma multiforme (GBM) is the most prevalent and aggressive primary malignant tumor of the central nervous system (CNS), with a median survival of 14.6 months and a 5-year survival rate of only 5.5% (1). Although comprehensive therapies combined chemotherapy and radiotherapy after surgery can prolong survival, the chemoresistance is one of the major causes of relapse as well as poor survival in GBM patients. Temozolomide (TMZ), an oral alkylating agent, is the first-line chemotherapeutic agent for GBM treatment in current (2). Nevertheless, the intratumoral hypoxic microenvironment caused by high invasiveness, vasculogenesis, and vascular malformation of GBM cells, further contributes to the dissatisfaction of the response rate of patients to TMZ-based chemotherapy and induces high recurrent rate of GBM. Therefore, it is of great significance to elucidate the potential molecular mechanisms related to the promotion of chemotherapeutic resistance in GBM hypoxic microenvironment for the development of new treatment strategies.

Multiple mechanisms are involved in tumor chemoresistance, including dysregulation of apoptosis, cancer stem cells, augmented DNA repair activities and ncRNAs. It has been revealed that microRNAs (miRNAs), such as miR-181b, miR-124, miR-26a (3–5), participated in GBM chemoresistance. The aberrant expression of hypoxia-regulated miRNAs plays key roles in GBM development, including cell proliferation, apoptosis, and invasion as well as sensitize to TMZ in GBM therapy (6–8). In previous studies, we found that the expressions of miR-26a and miR-137 in GBM cells after hypoxia treatment were significantly dysregulated, and hypoxia could induce the protective response to mitochondrion *via* HIF-1α-mediated miR-26a upregulation which was associated with TMZ resistance (5). Meanwhile, miR-137 has been reported as a tumor suppressor in a variety of tumors like cervical cancer, pancreatic cancer as well as astrocytoma (9–11). Another article also reported that lncRNA NCK1-AS1 could increase drug resistance of GBM cells to TMZ by modulating miR-137/TRIM24 (12), prompting that miR-137 may associate with GBM chemosensitivity. However, the mechanism of miR-137 regulating TMZ resistance under GBM hypoxic conditions has not been thoroughly elucidated.

LRP6 is a member of the expanding LDL receptor family which functions as an indispensable co-receptor in Wnt signaling pathway. LRP6 has been considered as the fundamental drivers of chemoresistance in hepatocellular carcinoma, which closely related to tumor recurrence (13). Recent research revealed that blocking the interaction between Wnt protein and their co-receptors such as LRP6 contributes to the anti-tumor effect in glioblastoma (14). Wnt/β-catenin signaling is a key signaling pathway, which regulates epithelial-mesenchymal transition (EMT) in cancers (15, 16). EMT is the major cause of invasion and metastasis. GBM arises from glial cells which originated in the ectodermal epithelium of the blastoderm, despite the brain is lacking critical tissue components (epithelium and mesenchyme), studies have found that key invasion signaling pathways overlap between CNS and other cancers, and EMT-related genes are also involved in proliferation, apoptosis, and invasion of GBM cells, and associated with GBM progression and chemoresistance (17). However, little information is known about the mechanism of miR-137 and LRP6/β-catenin signaling in regulation of EMT progression and TMZ chemo-sensitivity in GBM.

The aim of this study is to further understand the role and mechanism of miR-137 during hypoxia-induced TMZ resistance processes in GBM. We would address the following questions: (1) whether miR-137 acts as a tumor suppressor to affect cell invasion and EMT in GBM cells with TMZ treatment, (2) whether LRP6 is the direct target of miR-137, (3) whether miR-137/LRP6 is involved in regulating cell proliferation, apoptosis, invasion, and the expression levels of EMT-related genes in GBM, associated with TMZ chemosensitivity, (4) whether miR-137/LRP6 inhibits the tumor growth *in vivo*, (5) how the hypoxia regulated miR-137 expression. Our findings will provide further insight into the mechanism by which miRNAs-regulates chemotherapeutic resistance in the GBM hypoxic microenvironment.

## Methods

### Clinical Specimens

The human glioma tissue samples (n = 59) were obtained from patients undergoing standard surgical procedure at the Department of Neurosurgery of the First Affiliated Hospital of Nanjing Medical University, China. Normal brain tissues (n = 9) were collected as negative controls from patients undergoing decompressive craniectomy for traumatic brain injury. Each sample was frozen in liquid nitrogen immediately. The pathological diagnosis of glioma is confirmed by pathologists. The experiment has passed ethical review by the medical ethics committee of the First Affiliated Hospital of Nanjing Medical University (Ethics number: 2019-SR-479).

### Cell Culture and Reagents

Human glioblastoma cell lines U87 and U251 (purchased from the Chinese Academy of Sciences Cell Bank, Shanghai, China) were cultured in Dulbecco’s modified Eagle’s medium (DMEM) with 4.5 g/L D-glucose, L-glutamine, 110 mg/L sodium pyruvate (Gibco, USA), and added 10% fetal bovine serum (FBS), 100 units of penicillin/mL, and 100 ng of streptomycin/mL. Human embryonic kidney 293T (HEK-293T) (Chinese Academy of Sciences Cell Bank, Shanghai, China) cells were cultured in DMEM medium contained with 10% FBS, 100 units of penicillin/mL, 100 ng of streptomycin/mL, and 2 mmol/mL glutamine. Cells were incubated at 37°C in an atmosphere of 5% CO_2_ and did the mycoplasma contamination of cell lines regularly. The cells were cultured at 37°C in an atmosphere of 1% O_2_ atmosphere for hypoxia treatment. TMZ and 5-azacitidine were purchased from Sigma (USA). Antibodies against β-catenin (#8814), E-cadherin (#14472), and Ki-67(#9449) were purchased from Cell Signaling Technology (USA), LRP6 (ab134146, ab24386), N-cadherin (ab18203), Vimentin (ab8978), and 5-mC(ab214727) antibodies were purchased from Abcam (UK), and GAPDH (MB001) antibody was obtained from Bioworld (USA).

### Lentivirus Packaging and Establishment of Stable Cell Lines

The lentiviral packaging kit was purchased from Open Biosystems (Huntsville, AL, USA). Lentivirus carrying hsa-miR-137 or hsa-miR-negative control (miR-NC) was packaged in HEK-293T cells and collected from the media supernatant following the manufacturer’s manual. Stable cell lines were established by infecting lentiviral soup into U87 and/or U251 cells and followed by puromycin selection. U87 and U251 cells expressing miR-137 or miR-NC were infected by the lentiviral supernatant containing pReceiver-Lv-105-LRP6 or pReceiver-Lv-105-NC (Gene Copoeia, USA) according to the manufacturer’s instructions.

### shRNA Transfection

The sequence of shRNA targeting DNMT1 (DNMT1 shRNA) and scrambled negative control (NC shRNA) were purchased by Santa Cruz. U87 cells seeded in six-well plates were transfected DNMT1 shRNA or NC shRNA using Lipo-fectamine 3000 transfection reagent (Invitrogen, USA) according to the manufacturer’s instructions, and then cultured at 37°C in an atmosphere of 1% O_2_ for hypoxia treatment.

### RNA Extraction and qRT-PCR Analysis

Trizol reagent (Invitrogen, CA, USA) was used to extract the total RNAs from harvested cells and tissues according to the manufacturer′s instructions. Then the total RNAs were reversely transcribed by The PrimeScript RT Reagent Kit (Vazyme Biotech Co., Ltd, China) to determine the mRNA levels of LRP6. GAPDH was used as internal control. Primers used for qRT-PCR were as follows: LRP6 forward primer, 5′-TGTCAGCGAAGAAGCCATTAAA-3′, LRP6 reverse primer, 5′-TCTAAGGCAATAGCTCTGGGT-3′, GAPDH forward primer, 5′-CCACCCATGGCAAATTCCATGGCA-3′, GAPDH reverse primer, 5′-TCTAGACGGCAGGTCAGGTCCACC-3′. To measure the expression levels of miR-137, stem-loop specific primer method was used as described previously (18, 19). Expression of U6 was used as an endogenous control. Quantitative reverse transcriptase (qRT) PCR primers were the following: miR-137 RT primer, 5′- GTCGTATCCAGTGCAGGGTCCGAGGTATTCGCACTGGATACGACTGCCGC-3′; miR-137 PCR primers, sense: 5′-GCGAGCGAGGAGAGACCA-3′; antisense: 5′-AGTGCAGGGTCCGAGGTATT-3′. U6 RT primer: 5′-AACGCTTCACGAATTTGCGT-3′; U6 PCR primers sense: 5′-CTCGCTTCGGCAGCACA-3′; antisense: 5′-TGGTGTCGTGGAGTCG-3′. The cDNAs were amplified by ChamQ SYBR^®^ qPCR Master Mix (Vazyme Biotech co., ltd, China) on a LightCycler480II Real-Time PCR thermocycler (Roche, Switzerland), and fold changes were calculated by relative quantification (2^-△△Ct^) (20, 21).

### CpG Island Prediction

CpG islands were predicted using the program MethPrimer (22). The DNA sequences was input into MethPrimer, this program returned results of CpG island prediction.

### DNA Isolation and Methylation Analyses

A Quick-gDNA kit (Zymo Research, Irvine, CA, USA) was purchased to extracted genomic DNA, and genomic DNA (1 mg) was modified with sodium bisulfate using EpiTect Bisulfite kit (Qiagen, Valencia, CA). Methylated DNA immunoprecipitation (MeDIP assay) was performed using a MeDIP ChIP kit (Abcam, Cambridge, MA, USA) following the manufacturer’s instruction. Genomic DNA was extracted in normoxia/hypoxia treated U87 cells. The genomic DNA were sonicated to shear to 500 bp fragments and quantified by a spectrophotometer. The sheared genomic DNA fragments were incubated with the 5-Methylcytosine (5-mC) Rabbit mAb overnight at 4°C with rotation. Then, the complex was captured by ChIP-Grade Protein G Magnetic Beads with 2 h-incubation at room temperature. The DNA fragments were eluted from the beads. After purification, the immunoprecipitated DNA was quantified using a SYBR Green qPCR kit (Applied Biosystem, Foster City, CA, USA). The promoter sequence fragment of miR-137 containing the CpG island was input into Primer Premier 5 software and the primers were designed. The primers used were: Forward: 5′-AATGAGTGTTTATTTTCCATGATCT-3′. Reverse: 5′- TATGATTGAGTGCCATGGCGGCCAG-3′. The enrichment of miR-137 was presented as the percent of input with the formula: percent of INPUT = 10% × 2^(C[T] 10%Input Sample – C[T] IP Sample)^.

### Protein Extraction and Immunoblotting

Cells or tissues were harvested and lysed on ice in RIPA buffer (150 mM NaCl, 100 mM Tris, 10 mM sodium fluoride, 5 mM EDTA, 1% Triton X-100, 1% sodium deoxycholate, and 0.1% SDS), supplemented with 1 mM sodium orthovanadate, 2 mM leupeptin, 1 mM phenylmethylsulfonyl fluoride (PMSF), 2 mM DTT, and 2 mM pepstatin. The lysates were centrifugated at 12,000 rpm, 4°C for 15 min, the supernatants were harvested, and BCA assay (Beyotime, China) was performed to determine the protein concentrations. Protein extracts were separated by SDS-PAGE, and transferred to PVDF membranes (Roche, Switzerland) in transfer buffer [20 mM Tris, 150 mM glycine, 20% (v/v) methanol]. Membranes were blocked with 5% non-fat milk for 2 h and incubated with primary antibodies. The ECL Detection System (Thermo Scientific, Rockford, IL, USA) was used for signal detection.

### Dual-Luciferase Reporter Assay

The 3’-UTRs of LRP6, which contain predicted miR-137 seed-matching sites and corresponding mutant sites were synthesized and annealed, and inserted into the SacI and HindIII restriction enzyme sites of pMIR-REPORTER vector (Ambion, CA, USA), and were validated by sequencing. TOP-Flash/FOP-Flash reporter plasmids were purchased from Millipore (Merck Millipore, USA). U87 cells were seeded in a 24-well plate and co-transfected with the wild-type plasmid or mutated reporter plasmid, and miR-137 or miR-NC for Dual-luciferase reporter assay. Luciferase activities were analyzed 24 h after transfection using the Dual Luciferase Reporter Assay System (Promega, WI, USA).

### Immunofluorescence Assay

Cells transfected with miR-137 or miR-NC, or co-transfected LRP6 in the present of TMZ or DMSO after 48 h incubation were fixed with 4% formaldehyde, permeabilized with PBS containing 0.5% Triton X-100 at 4°C, and blocked with 1% bovine serum albumin (BSA) in PBS containing 0.1% Triton X-100. Cells were immunostained with antibodies against E-cadherin and Vimentin PBST containing 1% BSA overnight at 4°C. The antigen-primary antibody complex was detected using fluorescence isothiocyanate (FITC)-labeled goat anti-rabbit secondary antibodies (Life, Technologies, USA), and DAPI (Life, Technologies, USA) was used to detect nucleus. Microscopic observation was performed under a fluorescence microscope (Carl Zeiss Jena, Germany).

### Cell Proliferation Assay

Cells (3 × 10^3^) were seeded per well and cultured in 96-well plates. A CCK8 kit (Dojindo Laboratories, Kumamoto, Japan) was performed for cell proliferation assay according to the manufacturer’s instruction at indicated time points. Three independent experiments were performed in triplicate.

### Flow Cytometric Analysis

ANXA5 and PI were stained by the Alexa Fluor 488Annexin V Dead Cell Apoptosis Kit (BD Pharmingen) as manufacturer’s protocol. The cells were analyzed by flow cytometry (BD FACSCalibur, USA). Data was analyzed by FlowJo. Three independent experiments were performed in triplicate.

### Invasion Assay

Twenty-four-well BD Matrigel invasion chambers (BD Biosciences, Cowley, UK) were used for invasion assay according to the manufacturer’s instructions. Cells (5 × 10^4^) were seeded per well in the upper well of the invasion chamber in DMEM without serum, while the lower chamber well contained DMEM supplemented with 10% FBS to stimulate cell invasion. After incubation for 24 h, cells from the upper surface of Millipore membranes were removed, and the migrated cells on the lower surface of membranes were fixed and stained with 4% paraformaldehyde, stained with 0.1% crystal violet, and photographed in three independent 10× fields for each well. Membrane was air-dried and soaked for 15 min at room temperature with 33% acetic acid decolorization (200 µL/well). The destained solution was transferred to 96-well plates and read the absorbance value at OD570. Three independent experiments were conducted in triplicate.

### *In Situ* Tumor Formation Experiment

U87-Luc cells (5 × 10^5^) with indicated virus infection or treatment were resuspended in 100 µL serum-free DMEM and injected intracranially into the striatum of 6-week-old BALB/c nude mice (Vital River Animal Center, Beijing, China) by a stereotactic device (coordinates: 2 mm anterior, 2 mm lateral, 3 mm depth from the dura). One week later, the tumor-bearing mice were intraperitoneally injected with 20 mg/kg TMZ in saline. Mice received injected once a day for 5 consecutive days and stopped the injections for 2 days, corresponding to one cycle of TMZ treatment, continuing treatment for 4 weeks (17, 23, 24). To visualize cell proliferation, mice were performed intraperitoneal injection with D-luciferin (150 mg/kg) and formatted of image under anesthesia with the IVIS Illumina System (Caliper Life Sciences). Picture acquisition was conducted once a week to supervise tumor growth. After 3 or 4 weeks, brains were perfused with 4% paraformaldehyde by cardiac perfusion and further fixed with Bouin solution at 4°C overnight. Then brains were resected and performed H&E staining. Mice were killed to dissect the tumor tissues for further study.

### Immunohistochemistry Assay

Tumor tissues in mice were fixed with Bouin solution for 24 h, washed with 70% ethanol, and processed by the paraffin-embedded method. Tissue slides (5 μm thick) were heat immobilized or pepsin immobilized and incubated with antibody against LRP6 and Ki-67 overnight at 4°C. After incubated with secondary antibody, the slides were reacted with DAB Histochemistry Kit (ZSGB-BIO, China).

### Statistics Analysis

All data were expressed as means ± standard deviation (SD) from at least three independent experiments except those specifically indicated. Student’s t test was used for comparison between two groups and one-way analysis of variance (ANOVA) was applied to multiple group comparison. The Kaplan–Meier method with the logrank test was used to calculate the overall survival (OS) rate for comparison between different groups. The correlations between variables were analyzed with the Spearman correlation coefficient. Data were analyzed using GraphPad Prism 7 software (La Jolla, USA) or IBM SPSS Statistics 23.0 software (Chicago, IL, USA). A *p*-value <0.05 was considered as statistically significant.

## Results

### MiR-137 Downregulation Correlates to TMZ Resistance

**Figure 1:**
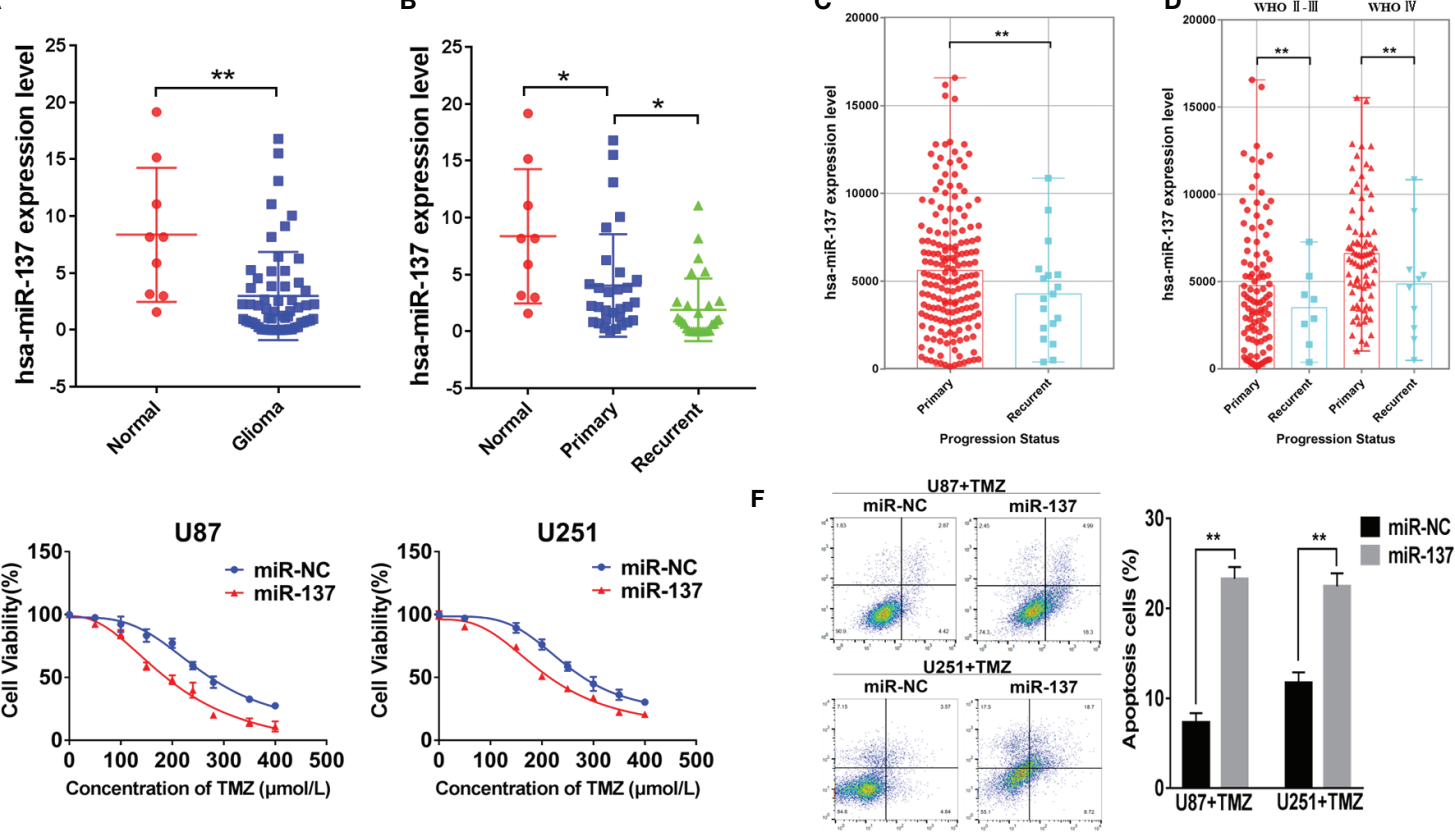
MiR-137 downregulation correlates to TMZ resistance. **(A)** Relative expression of miR-137 in normal brain tissues and GBM tissues. The miR-137 expression was normalized to U6. **(B)** Relative expression of miR-137 in primary and recurrent GBM tissues, normal brain tissues. The miR-137 expression was normalized to U6. **(C)** The expression of miR-137 in primary and recurrent glioma patient samples in the CGGA. **(D)** The expression of miR-137 in different grades of primary and recurrent glioma patient samples in the CGGA. **(E)** CCK-8 showing viability of U87 and U251 cells stably expressing miR-137 or miR-NC with TMZ treatments at different concentrations for 72 h. **(F)** Flow cytometric analysis revealing the effect of miR-137 overexpression on the apoptosis of U87 and U251 cells with TMZ treatment (200 μM, 48 h). Data are representative by means ± SD in triple experiments. Significant results were presented as *P < 0.05 and **P < 0.01.

We used qRT-PCR to measure the expressions of miR-137 in clinical specimens (9 normal brain tissues and 59 GBM tissues) and determined that miR-137 were lower-expression in GBM tissue samples compared with normal brain tissues (**Figure 1A**). Furthermore, we subdivided the 59 samples into primary and recurrent GBM and found that the recurrent GBM displayed the lowest miR-137 expression levels (**Figure 1B**), indicating that miR-137 may be related to the prognosis of GBM. Then, we searched CGGA (Chinese Glioma Genome Atlas) database to grasp the expression of miR-137 in different progression status of primary and recurrent glioma, the results were verified our previous study, miR-137 showed lower expression levels in recurring gliomas than those in primary gliomas in the different WHO grades (**Figures 1C, D**).

The prognosis of GBM was closely related to the chemotherapy resistance. In order to determine whether miR-137 had function on GBM cell chemoresistance to TMZ, we firstly constructed two GBM cell lines that expressed miR-137 stably (**Figure S1A**), then miR-137-overexpressing cells treated with TMZ were used to analyze cell viability and apoptosis. The results showed that cells overexpressed miR-137 were more sensitive to TMZ (**Figures 1E, F**). These data adumbrated that miR-137 was correlated with GBM cell chemoresistance to TMZ thus affecting the prognosis of GBM.

### Ectopic Expression of miR-137 Inhibits Invasion and EMT in GBM Cells With TMZ Treatment

**Figure 2:**
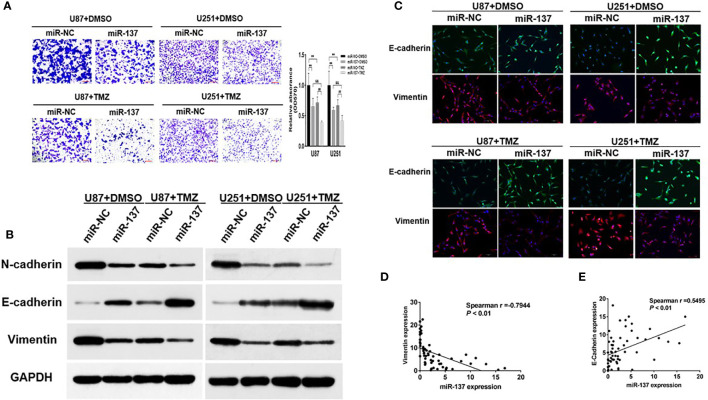
Ectopic expression of miR-137 inhibits invasion and EMT in GBM cells with TMZ treatment. **(A)** Invasion assays of U87 and U251 cells stably expressing miR-137 or miR-NC with TMZ treatments (200 μM, 24 h). Scale bar = 100 μm. **(B)** Western blot analysis of N-cadherin, E-cadherin, and Vimentin in U87 and U251 cells stably expressing miR-137 or miR-NC with TMZ treatments (200 μM, 48 h). GAPDH was used as internal control. **(C)** U87 and U251 cells transduced with miR-NC or miR-137 with TMZ treatment (200 μM) for 24 h were stained with E-cadherin and Vimentin and imaged by fluorescent microscope (20× field). **(D)** Spearman′s correlation analysis determined the correlation between expression levels of Vimentin and miR-137 in human GBM specimens. **(E)** Spearman′s correlation analysis determined the correlation between expression levels of E-cadherin and miR-137 in human GBM specimens. Data are representative by means ± SD in triple experiments. ** indicates significant difference compared to miR-NC+DMSO group at *p* < 0.01; ^##^ indicates significant difference compared to miR-NC+TMZ group at *p* < 0.01, ^&&^ indicates significant difference compared to miR-137+DMSO group at *p* < 0.01.

The capability of cell invasion is a critical progression in tumor malignancy and recurrent. In **Figure 2A**, we found that compared with U87 and U251 cells expressing miR-137, the combined effect of miR-137 and TMZ treatment significantly inhibited cell invasion. EMT has been recognized as an important factor in tumor invasion and chemoresistance, we wondered whether a set of protein markers associated with EMT was changed. Then, we tested the protein levels of EMT-related markers, such as N-cadherin, E-cadherin, and Vimentin by immunoblotting assay. Our results showed that the expression levels of E-cadherin were upregulated in miR-137-overexpressing GBM cells, compared with negative control, whereas N-cadherin and Vimentin protein levels were decreased. Meanwhile, the combined effect of miR-137 and TMZ treatment were further enhanced (**Figure 2B** and **Figure S2A**). And similar results were obtained by the immunofluorescence assay (**Figure 2C**). Further analysis of the relationship between miR-137 and Vimentin or E-cadherin in clinical tissue samples showed that miR-137 and Vimentin was negatively correlated (Spearman r = −0.7944), while miR-137 was positively correlated with E-cadherin (Spearman r = 0.5495) (**Figures 2D, E**), indicating that miR-137 may inhibit the tumor invasion by affecting EMT-related factors. Collectively, these results suggested that ectopic expression of miR-137 could suppress invasion and EMT in TMZ-treated GBM cells *in vitro*.

### MiR-137 Directly Targeted LRP6

**Figure 3:**
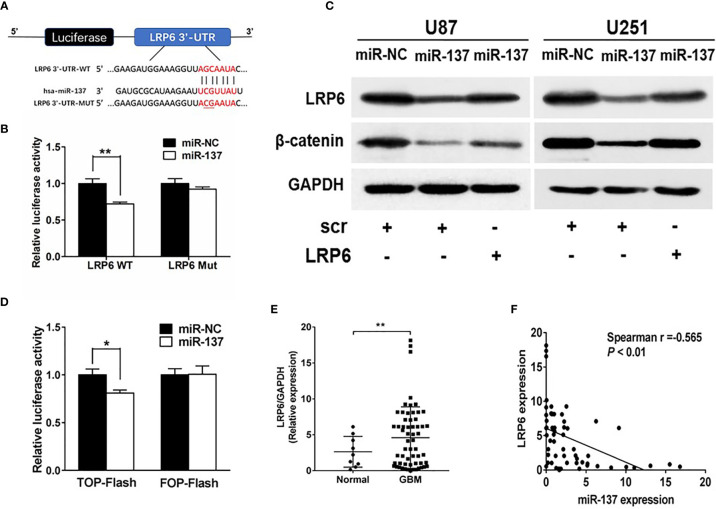
LRP6 is a direct target of miR-137. **(A)** The complementary pairing of miR-137 with LRP6 wild-type (WT) and mutant (Mut) 3′-UTR reporter constructs were shown. **(B)** The reporter plasmids carrying the WT or Mut LRP6 3′-UTR regions were co-transfected with miR-137 or miR-NC and pRL-TK into 293T cells. After 24 h of the transfection, the relative luciferase activities were analyzed. **(C)** U87 and U251 cells were transfected with miR-137 or miR-NC followed by LRP6 transfection. LRP6 and β-catenin expression levels were determined by immunoblotting, GAPDH as an internal control. **(D)** Relative luciferase activity in U87 cells co-transfected with miR-NC or miR-137 and the reporter vector TOP-Flash or its mutant FOP-Flash. Luciferase assay was performed to determine the transcriptional activity of β-catenin. **(E)** The expression levels of LRP6 in normal brain and GBM tissues. **(F)** Spearman′s correlation analysis determined the correlation between expression levels of LRP6 and miR-137 in human GBM specimens. Data are representative by means ± SD in triple experiments. Significant results were presented as *P < 0.05, **P < 0.01.

To further understand potential role and mechanism how miR-137 affects the EMT-related genes, TargetScan was used to predict potential targets of miR-137. We found that LRP6, recognized as a co-receptor to facilitate Wnt/β-catenin pathway by promoting β-catenin nuclear translocation, could be a potential target of miR-137. The putative binding sites in 3’-UTR regions of LRP6 for miR-137 were shown in **Figure 3A**. Next, the binding-site luciferase reporter assay was performed to verify whether miR-137 directly targeted LRP6. At first, we verified the efficiency of miR-137 overexpression by qRT-PCR (**Figure S1B**). Then 293T cells were co-transfected with the wild type (WT) or mutated (Mut) LRP6 luciferase reporter plasmid together with miR-137 or miR-NC, and the luciferase activities were measured after 24 h. As shown in **Figure 3B**, overexpression of miR-137 significantly reduced luciferase activities of wild type LRP6 3’-UTR reporter to 70%, but not that of mutant reporter, suggesting that miR-137 may inhibit the 3’-UTR function of LRP6 through binding to the seed sequence. The protein expression levels of LRP6 and β-catenin were decreased in miR-137-overexpresing cells (**Figure 3C** and **Figure S2B**). Further, forced expression of LRP6 without 3’-UTR regions in miR-137-overexpressing cells partially restored LRP6 and β-catenin expression, indicating that miR-137 probably inhibit β-catenin expression *via* LRP6 (**Figure 3C** and **Figure S2B**). We used TOP-Flash and FOP-Flash reporter assays to detect the transcriptional activity of β-catenin in cells. miR-137 reduced TOP-Flash luciferase activity to 80% (**Figure 3D**). These results suggested that miR-137 could regulate β-catenin and its downstream signaling pathways by LRP6.

To further determine the clinical correlation between miR-137 and LRP6 levels, we selected 9 normal brain tissues and 59 GBM tissue samples to explore the relevance of miR-137 and LRP6 levels. As shown in **Figure 3E**, LRP6 expression levels were increased in GBM tissues compared with normal brain tissues. The spearman’s correlation analysis of glioblastoma specimens demonstrated that LRP6 levels were inversely correlated with miR-137 expression levels in GBM samples (Spearman r = −0.565) (**Figure 3F**). Taken together, we demonstrated that LRP6 was a direct target of miR-137 in both GBM cells and tissues.

### Overexpression of LRP6 Partially Reversed the Inhibitory Effect of miR-137

**Figure 4:**
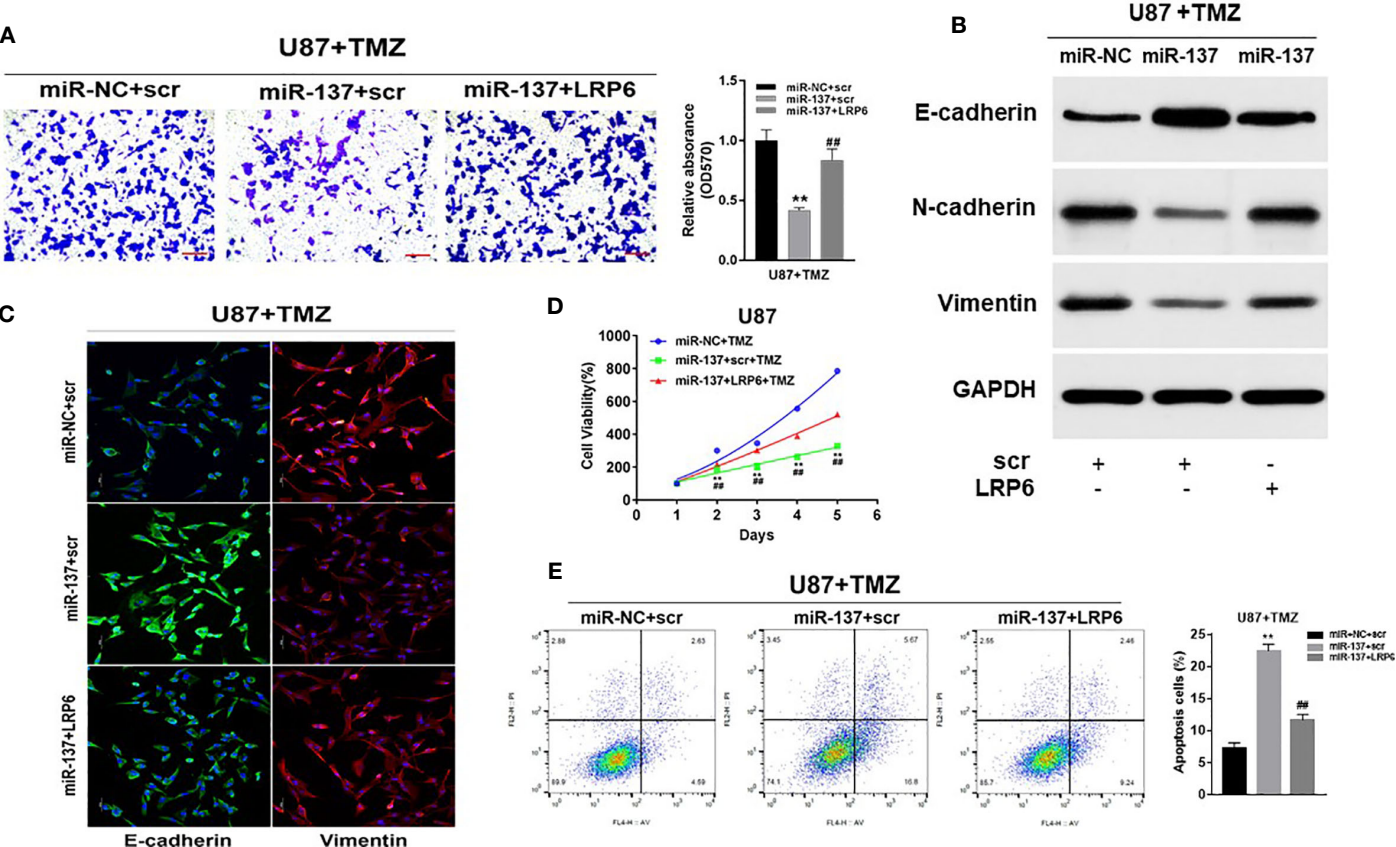
Overexpression of LRP6 partially reversed the inhibitory effect of miR-137. U87 cells stably expressing miR-137 or miR-NC, with or without overexpression of LRP6. Cells were pretreated with TMZ treatments (200 μM). **(A)** Cell invasion was evaluated by transwell assay. Scale bar = 100 μm. **(B)** N-cadherin, E-cadherin and Vimentin expression levels were determined by immunoblotting using GAPDH as an internal control. **(C)** Cells transduced with miR-NC or miR-137 with TMZ (200 μM) treatment for 24 h, with or without overexpression of LRP6, were stained with E-cadherin and Vimentin and imaged by fluorescent microscope (20× field). **(D)** Cell viability was tested every 24 h for different times with treatment of 200 μM TMZ. **(E)** Cell apoptosis was evaluated by flow cytometry assay. Data are representative by means ± SD in triple experiments. Significant results were presented as ^**^ *P* < 0.01, compared with miR-NC+TMZ group; ^##^ *P* < 0.01, compared with miR-137 group.

To further study whether miR-137 regulated GBM cell biological functions by targeting LRP6, we found that forced expression of LRP6 without 3’-UTR regions rescued the effect of miR-137 on cell invasion with TMZ treatment (**Figure 4A**). Meanwhile, overexpression of LRP6 restored miR-137 overexpression-attenuated N-cadherin and Vimentin, and decreased E-cadherin expression induced by miR-137 in TMZ-treated U87 cells (**Figure 4B** and **Figure S2C**). It showed similar results in immunofluorescence assay (**Figure 4C**). Moreover, CCK8 proliferation assay revealed that LRP6 partially abolished miR-137-suppressed cell resistance to TMZ (**Figure 4D**). Also, miR-137 promoted TMZ-induced apoptosis was suppressed by LRP6 (**Figure 4E**). Thus, we discovered that miR-137/LRP6 axis contributes to U87 cell chemosensitivity to TMZ by influencing cell growth, invasion, and apoptosis.

### MiR-137/LRP6 Axis Contributes to TMZ Sensitivity *In Vivo*

**Figure 5:**
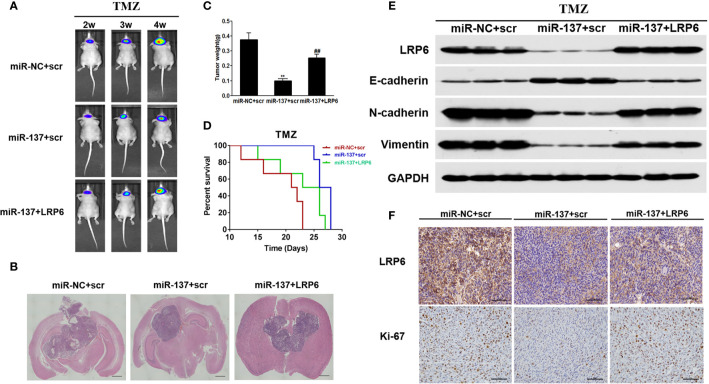
MiR-137/LRP6 axis contributes to TMZ sensitivity *in vivo*. **(A)** U87-Luc/miR-137, U87-Luc/miR-NC, or U87-Luc/miR-137+LRP6 (5 × 10^5^ cells) were dispersed in 100 μl of serum-free DMEM medium and implanted intracranially in each mouse (n = 8). Representative pseudocolor bioluminescence images of intracranial mice bearing miR-NC, miR-137, or miR-137+LRP6 transduced U87-Luc cells after treatment with TMZ (20 mg/kg) at indicated time points. **(B)** Intracranial tumors were shown by H&E staining. Scale bar = 1 mm. **(C)** The tumors were excised and weighed after 28 days. Data are representative by means ± SD in triple experiments. ** indicates significant difference compared to the miR-NC group at *P* < 0.01; ^##^ indicates significant difference compared to the miR-137 group at *P* < 0.01. **(D)** Kaplan-Meier survival curves of nude mice were shown. **(E)** The total proteins were extracted from xenografts and subjected to Western blot analysis for LRP6, N-cadherin, E-cadherin, and Vimentin expression. GAPDH expression level was served as an internal control. **(F)** The expression of LRP6 and Ki67 were analyzed in tumor tissues by immunohistochemistry (Scale bar = 50 μm).

*In situ* tumor formation experiment was performed in order to determine roles of miR-137/LRP6 pathway in regulating tumor chemosensitivity to TMZ *in vivo*. The results showed that the size of intracranial tumors in miR-137 group was much smaller than that in miR-NC control group after days of implantation, whereas miR-137+LRP6 group showed bigger tumor size after 4 weeks TMZ treatment (**Figures 5A, B**). In agreement with the tumor formation, the weight of tumors from miR-137 overexpression group were significantly smaller than those from the miR-NC overexpression group, while the tumor size from miR-137+LRP6-overexpression group was much bigger than that from miR-137 overexpression group (**Figure 5C**). In addition, mice with high miR-137 expression also showed the longest survival time in three groups, while LRP6 shortened their lifetime (**Figure 5D**). Meanwhile, in agreement with *in vitro* studies, protein expression levels of LRP6 were downregulated in miR-137-overexpressing group. Furthermore, the expression levels of N-cadherin and Vimentin were significantly suppressed in miR-137 group, while E-cadherin was enhanced by miR-137 overexpression. Forced expression of LRP6 without its 3’-UTR restored these protein expression levels regulated by miR-137 in tumor tissues (**Figure 5E** and **Figure S2D**). Immunohistochemistry assay results of LRP6 and Ki-67 expressions in three groups also showed that LRP6 could reverse the inhibited ability of tumor proliferation by miR-137 *in vivo* (**Figure 5F** and **Figure S3**). These results indicated that miR-137/LRP6 axis contributes to TMZ sensitivity *in vivo*.

### Mechanism of miR-137 Inhibition in GBM

**Figure 6:**
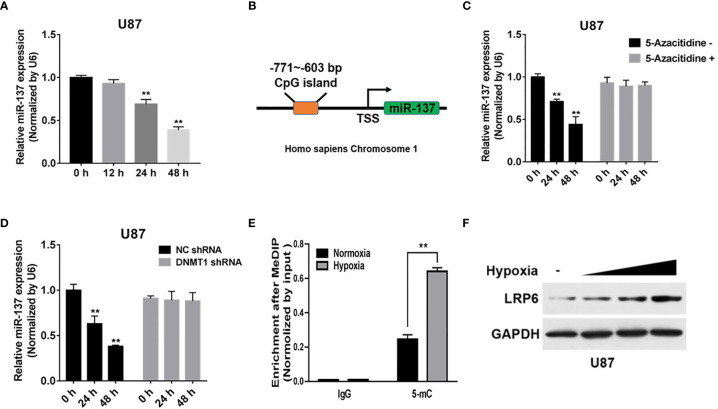
Hypoxia-induced miR-137 methylation inhibited the expression of miR-137 in GBM. **(A)** qRT-PCR was performed to measure time-dependent expression levels of miR-137 after hypoxia-exposure. **(B)** The CpG island in the promoter region of miR-137 was shown. **(C)** U87 cells were treated with or without 5-Aza-dC treatment for 0, 24, 48 h under hypoxic environment. miR-137 expression levels were measured by qRT-PCR. **(D)** U87 cells were transfected with DNMT1 shRNA and NC shRNA for 0, 24, 48 h under hypoxic environment, and using qRT-PCR to test miR-137 levels. **(E)** The enrichment of 5-mC was presented. **(F)** Immunoblotting results of LRP6 in U87 cells under after hypoxia-exposure. GAPDH expression level was served as an internal control. Data are representative by means ± SD in triple experiments. Significant results were presented as **P < 0.01.

Hypoxia is the common condition in most of the advanced solid tumors, which probably causes tumor suppressor miRNAs expression disorder and chemotherapy resistance (25). However, the association between the attenuation of miR-137 expression and hypoxia microenvironment in GBM remain unclear. Our results showed that the expression of miR-137, in GBM cells, was downregulated after hypoxia exposure in a time-dependent manner (**Figure 6A**). Meanwhile, the bioinformatic method prompts there existing a CpG island in the promoter region of miR-137 (**Figure 6B**). Existing research have reported that hypoxia has been shown to alter DNA methylation affecting genes expression, which means miR-137 is probably controlled by DNA methylation induced by the hypoxia stress. To confirm that, U87 cells were treated with 5-azacitidine or transfected with DNMT1 shRNA with hypoxia treatment. The results indicated that the inhibition of DNA methyltransferase may reduce hypermethylation of the miR-137 promoter and thus promote its re-expression, in the hypoxia condition (**Figures 6C, D**). To further explore if the DNA methylation influences miR-137 through the predicted CpG island, MeDIP was performed to check the enrichment status of methylated cytosine within the CpG island. Consistent with our hypothesis, the amount of methylated DNA which was pulled down by 5-mC antibody significantly increased in hypoxia-treated cells (**Figure 6E**). In addition, as the target molecule, the expression of LRP6 was increased in according with the time of hypoxia exposure, just the opposite of miR-137 expression (**Figure 6F** and **Figure S2E**). Collectively, hypoxia-induced DNA methylation resulted in the downregulation of miR-137 expression in GBM cells.

## Discussion

In previous studies, miR-137 was showed to be downregulated in human glioma tissues, which acted as a tumor suppressor to inhibit tumor growth and angiogenesis by directly targeting EZH2, EGFR, PTP4A3 (26–28). MiR-137 was also been reported involved in the sensitivity to chemotherapy, such as pancreatic cancer and ovarian cancer (29, 30) In this study, we found that miR-137 has low expression in GBM tissues, particularly in the recurrent GBM tissues. Moreover, overexpression of miR-137 inhibited cell viability and promoted cell apoptosis with TMZ treatment. These results revealed that miR-137 has considerable potential in the prognosis and to overcome TMZ resistance treatment of GBM patients. Chemotherapy is a major treatment modality for GBM. However, chemoresistance is a clinical problem that compromises the efficiency of treatment and ultimately results in treatment failure. The mechanisms by which miRNAs participate in drug resistance are complex. Studies have found that the molecular mechanisms of chemoresistance include EMT, DNA repair, autophagy, oncogenes, tumor suppressor genes, transporter pumps, mitochondrial changes, tumor Stemness, and exosomes (31). Hu YiRen et al. showed that miR-23b-3p was isolated by MALAT1, making it deviate from its target ATG12, which promoted autophagy and helped cancer cells escape lethal cell damage, thus contributing to autophagy-associated chemoresistance (32). Recent studies have demonstrated chemopreventive agents potentially promote immune evasion by upregulation of PD-L1 expression in cancer cells, thereby reducing tumor-specific T-cell activity (33). In our previous study, we demonstrated that miR-26a promotes TMZ resistance of glioma cells by inhibiting apoptosis and protecting mitochondrial membrane integrity. However, how miR-137 involvement in the process of GBM resistance to TMZ chemoresistance hasn’t been deeply researched.

EMT is associated with chemotherapy drugs resistance, resulting in recurrence and metastasis after standard regimen treatments in various cancers. Recently, several publications have shown how miR-137 attenuates EMT in different cancers. For example, miR-137 could inhibit cervical cancer cell invasion, migration and EMT by suppressing the TGF-β/smad pathway *via* binding to GREM1 (9). MiR-137 also was demonstrated that could inhibit BMP7 to enhance the EMT of breast cancer cells (34). Consistent with these results, we found that a hallmark of the EMT process- E-cadherin protein levels were increased, whereas N-cadherin and Vimentin protein levels were decreased in miR-137-overexpressing GBM cells with TMZ treatment. Although obvious morphological changes have not been observed, which may be due to the relatively short time of miR-137 transfection and TMZ treatment. And some studies have found that morphological changes occurred in drug-resistant cell lines with long-term TMZ processing, which still needs further study (17). Correlation analysis of clinical samples shown that miR-137 was positively correlated with E-cadherin, and contrary to Vimentin, indicating that miR-137 may promote TMZ sensitivity by affecting EMT-related factors. Accumulating evidence showed that Wnt/β-catenin pathway played crucial role in regulating EMT (35–37). Wnt/β-catenin pathway involves the stabilization of β-catenin through the binding of Wnt ligand to cell surface receptors such as LRP6. As a member of the low-density lipoprotein receptor gene family, LRP6 has been recognized as an oncogene, which can be regulated by miRNAs in glioma (38, 39). Our present study demonstrated that overexpression of miR-137 inhibited the expression of LRP6 and β-catenin. Cell invasion and the expressions of EMT-related molecules were affected by miR-137/LRP6 in the presence of TMZ. Furthermore, miR-137 enhanced the TMZ chemosensitivity through inhibiting GBM cell proliferation and promoting cell apoptosis by targeting LRP6. Taken these together, we demonstrated that miR-137 could effectively inhibit GBM growth and TMZ resistance through LRP6/β-catenin pathway.

Intratumoral hypoxia is a common phenomenon in human cancers (40), which could affect tumor progression, metastasis, and chemotherapy resistance (41). Recent reports also demonstrated that intratumoral hypoxia-induced miRNAs imbalance was associated with tumor cell proliferation, apoptosis, as well as TMZ resistance (5, 42, 43). In our previous study, we already demonstrated that hypoxia improved tolerance of GBM cells to TMZ and promoted tumor cells survival. miRNAs were dysregulation in the hypoxic environment and involved in TMZ resistance of glioma cells. Particularly, miR-137 was found to be downregulated under hypoxic conditions (5). In this paper, we further found the expression of miR-137 was obviously decreased under hypoxia in a time-dependent manner. It was reasonable to infer that the low expression of miR-137 might be caused by the ubiquitous hypoxic-microenvironment in GBM. Hypoxia has been shown to alter DNA methylation affecting genes expression (44). Approximately 20% of miRNAs were embedded within CpG islands, including miR-137, and was frequently silenced by methylation, thus inducing the miRNAs-expression inhibition in several tumors (45, 46). Here, our results also proved that inhibition of DNA methyltransferase reduced hypermethylation of the miR-137 promoter and promoted its re-expression in GBM hypoxia condition.

Overall, the present study demonstrated that miR-137 downregulation was induced by hypoxia, which could effectively regulate GBM growth and TMZ resistance through LRP6/β-catenin pathway. Our study significantly broadens the understanding of miR-137 in chemotherapy resistance. It is interesting to evaluate whether targeting miR-137 may have potential value as an adjuvant therapy in the future.

## Data Availability Statement

The original contributions presented in the study are included in the article/**Supplementary Material**, further inquiries can be directed to the corresponding authors.

## Ethics Statement

The studies involving human participants were reviewed and approved by the medical ethics committee of the First Affiliated Hospital of Nanjing Medical University. The patients/participants provided their written informed consent to participate in this study. The animal study was reviewed and approved by the Institutional Committee on Animal Care of Guangxi Institute of Chinese Medicine & Pharmaceutical Science.

## Author Contributions

D-ML and Q-DC mainly did the experiment and wrote the paper. G-NW and J-XY designed and conducted the animal experiment. JW and J-HH contributed to data analysis. XG and Z-MS mainly constructed the idea of this article and provided administrative, technical, and material support. All authors contributed to the article and approved the submitted version.

## Funding

This work was supported in part by National Natural Science Foundation of China (81772951, 81972610, 81703769, 82002914, 81772673, 81960729), Guangxi Science and Technology Project (AD18216002, AA17202040), Natural Science Foundation of Guangxi (2018GXNSFDA281046), Guangxi traditional Chinese medicine key discipline construction project (GZxk-z-20-75), the Priority Academic Program Development of Jiangsu Higher Education Institutions (PAPD).

## Conflict of Interest

The authors declare that the research was conducted in the absence of any commercial or financial relationships that could be construed as a potential conflict of interest.

## Abbreviations

miRNAs, microRNAs; LPR6, low-density lipoprotein receptor-related protein 6; EMT, epithelial-mesenchymal transition; WT, wild type; Mut, mutant; NC, negative control.
