# Supplementary material for: Hypoxia-Induced miR-137 Inhibition Increased Glioblastoma Multiforme Growth and Chemoresistance Through LRP6
Source: Front Oncol. 2021 Feb 25;10:611699. doi: 10.3389/fonc.2020.611699 (PMC7946983; doi:10.3389/fonc.2020.611699)

**Supplementary material**

**Hypoxia-induced miR-137 inhibition increased glioblastoma multiforme growth and** **chemoresistance through LRP6**

Dong-Mei Li, Qiu-Dan Chen, Gui-ning Wei, Jie Wei, Jian-Xing Yin, Jun-hui He, Xin Ge, Zhu-Mei Shi

**Supplementary Fig.1**

(a) qRT-PCR analysis of the expression of miR-137. U6 RNA served as the loading control. (b) qRT-PCR analysis of transfection efficiency of miR-137 overexpression. ** indicates significant difference compared to miR-NC at *p* < 0.01.


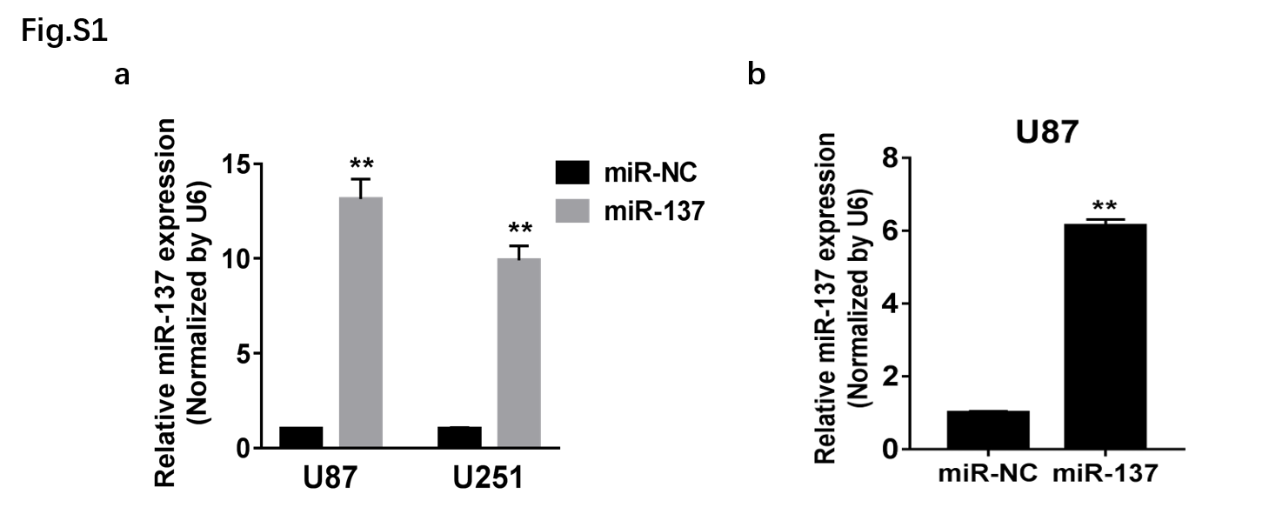


**Supplementary Fig.2**

(a) The relative density of N-cadherin, E-cadherin, Vimentin were detected by ImageJ software. ** indicates significant difference compared to miR-NC+DMSO group at *p* < 0.01; ## indicate significant difference compared to miR-NC+TMZ group at *p* < 0.01, && indicate significant difference compared to miR-137+DMSO group at *p* < 0.01. (b) The relative density of LRP6 and β-catenin in U87 and U251 cells. ** indicates significant difference compared to miR-NC+scr group at *p* < 0.01; ## indicate significant difference compared to miR-137+scr group at *p* < 0.01. (c) Density analysis of N-cadherin, E-cadherin, Vimentin in TMZ-treated U87 cells. ** indicates significant difference compared to miR-NC+scr group at *p* < 0.01; ## indicate significant difference compared to miR-137+scr group at *p* < 0.01. (d) Density analysis of N-cadherin, E-cadherin, Vimentin in tissues. ** indicates significant difference compared to miR-NC group at *p* < 0.01; ## indicate significant difference compared to miR-137 group at *p* < 0.01. (e) Relative density of LRP6 expression in U87 cells with TMZ treatment for different times. ** indicates significant difference compared untreated group at *p* < 0.01.


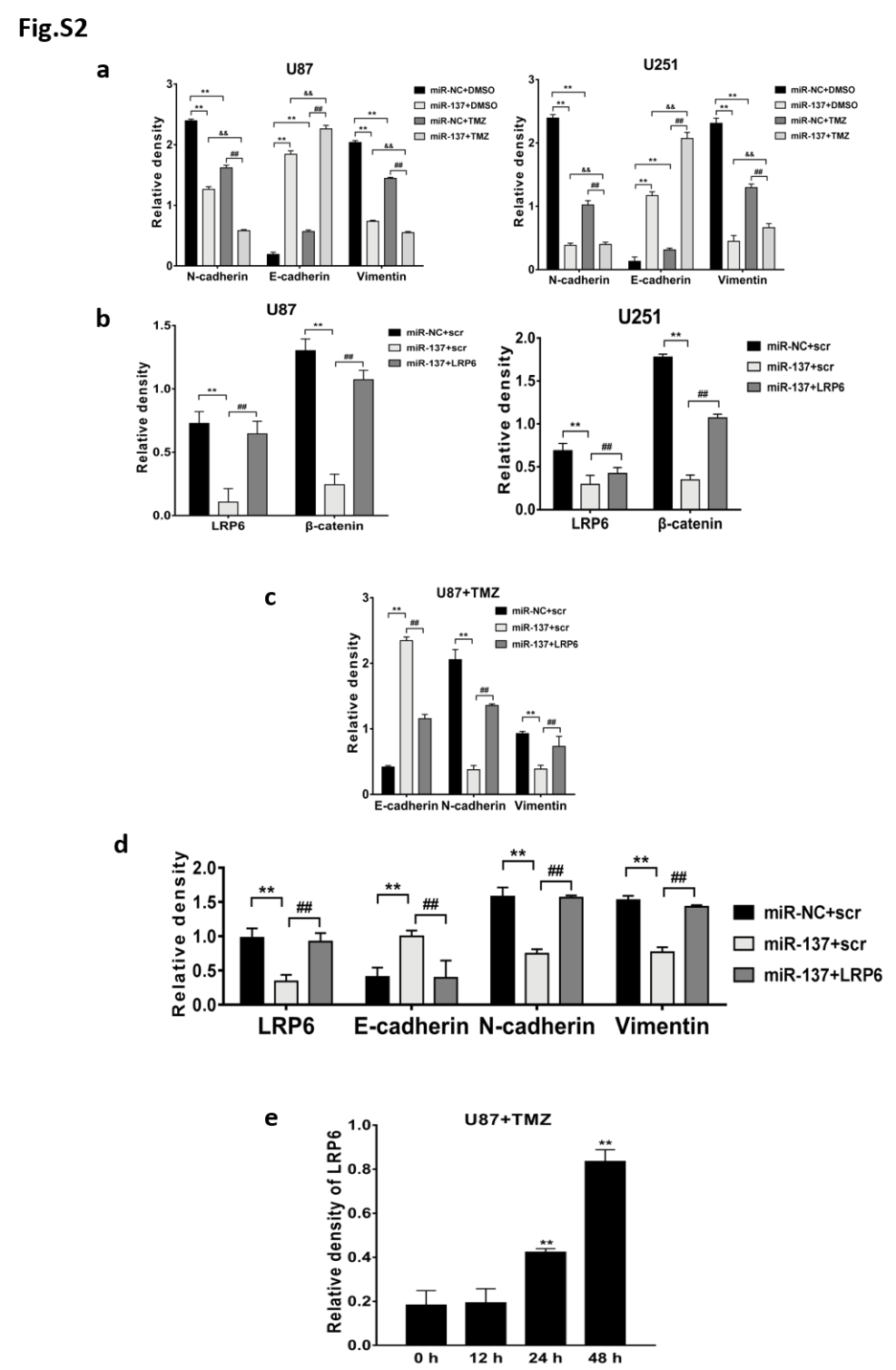


**Supplementary Fig.3**

The percentages of LRP6 and Ki-67 positive areas in tissue slices were analyzed by ImageJ software. ** indicates p < 0.01, compared to the control group.


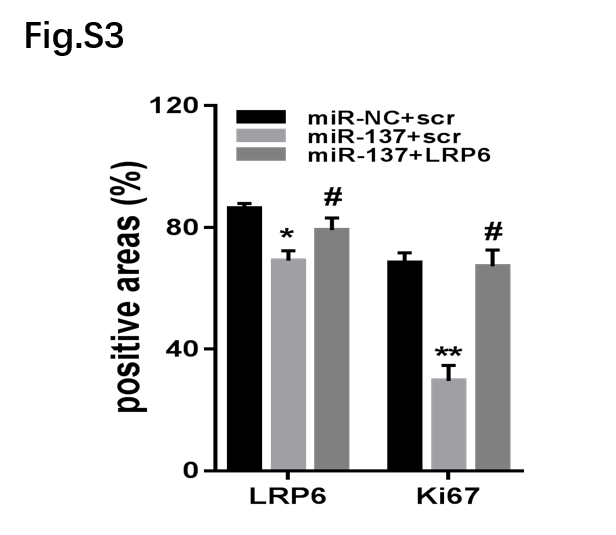

Supplement: Supplementary file 1 [file DataSheet_1.docx]
